# Treatment of chest wall tuberculosis with transdermal ultrasound-mediated drug delivery

**DOI:** 10.3892/etm.2015.2219

**Published:** 2015-01-27

**Authors:** YI HAN, QIUYUE ZHAO, DAPING YU, ZHIDONG LIU

**Affiliations:** Second Department of Thoracic Surgery, Beijing Chest Hospital, Capital Medical University, Beijing 101149, P.R. China

**Keywords:** chest wall tuberculosis, pulmonary tuberculosis, transdermal ultrasound-mediated drug delivery, cure rate, efficiency rate, recurrence rate

## Abstract

Chest wall tuberculosis (TB) is an endemic disease with a large number of variants. The condition affects numerous parts of the body and can penetrate the skin to form chronic open ulcers. Current treatment methods include oral anti-TB drugs and surgery. However, conventional drug treatments are not effective due to the difficulty in achieving an effective local concentration, and certain patients are unable to tolerate surgery. The recurrence rate for chest wall TB is high following surgery, and may result in the prolonged healing of wounds in certain patients, as well as chronic sinusitis and fistula formation. To identify a safe, simple, less invasive and more clinically effective treatment method, the present study investigated transdermal ultrasound-mediated anti-TB drug delivery. A total of 186 patients were selected and randomly divided into transdermal ultrasound, surgery and oral anti-TB drug only groups. Rifampicin was the drug delivered by transdermal ultrasound. The cure and efficiency rates were shown to be 87.10 and 93.55%, respectively, in the ultrasound treatment group. No statistically significant difference was observed in the cure rates between the transdermal ultrasound and surgery groups; however, a statistically significant difference was identified in the cure rates between the transdermal ultrasound and oral anti-TB drug only groups. Therefore, transdermal ultrasound technology was shown to deliver anti-TB drugs quickly and directly, which resulted in a high local concentration of the drug, overcoming the problem of obtaining an effective local drug concentration. The observations demonstrated that transdermal ultrasound-mediated drug delivery is an effective method by which to control TB, particularly when compared with traditional oral anti-TB therapy and surgery.

## Introduction

Extrapulmonary tuberculosis (TB) refers to TB involving the extrapulmonary organs of the body. Extrapulmonary TB accounts for 9.7–11.9% of all TB cases in China, while the mortality rate caused by extrapulmonary TB accounts for 14.1–17.6% of the total TB mortalities (1). A variety of signs and symptoms occur depending on the different lesion sites. The lesion sites in extrapulmonary TB are scattered and the detection rate of *Mycobacterium tuberculosis* (*M. tuberculosis*) in the lesions is low. Therefore, the condition of the patients with extrapulmonary TB is complicated and frequently overshadowed by other illnesses, thereby having a big impact on diagnosis and treatment. Functional lesions in local organs often cause disabilities and even endanger lives due to missed diagnosis, misdiagnosis and the lack of normative guidance on treatment protocols (2).

Chest wall TB is a common form of TB on the surface of the body, and includes TB lesions found in the ribs, sternum and soft tissue of the chest wall, which are secondary to pulmonary or pleural TB infection. The area of the lesions is large and the majority of lesions eventually appear as cold abscesses and ulcers, which lead to sinus tract or ulcer-induced fistulas (3). When a diagnosis is clinically confirmed, the abscesses are already formed and even ruptured, or the sinuses are formed. Treatment methods comprise oral anti-TB therapy combined with surgery. Although the local blood vessels of the TB lesions are damaged, the lymph nodes have intact capsules that are difficult for the TB drugs to penetrate, and a number of tubercle bacilli are contained within the lymph nodes (4). As the systemic anti-TB drugs are unable to penetrate into the lymph nodes, it is difficult for the tissue drug concentration to reach the effective bactericidal concentration required. Following oral administration of anti-TB drugs, achieving an effective concentration of the drug in the local lesions is difficult; thus, the intended antibacterial effects and sterilization cannot be realized. In addition, surgical treatment may cause large trauma, and requires combined intravenous-inhalation anesthesia. Certain patients are unable to tolerate these conditions, and even if the surgery is a success, the patient is left with a large surgical scar (5).

Sonophoresis technology is a type of transdermal-targeted drug delivery technology, whereby the drugs penetrate the tissue through the skin under the action of ultrasonic waves (6). The nature of biological tissues is that of a closed circuit, and sonophoresis applies high-frequency electromagnetic fields to produce a cavitation effect, which causes the arrangement of the stratum corneum lipid bilayer to become disordered and promotes transdermal drug penetration. A superimposed effect of multiple technologies is achieved through system integration, in order for the programed targeted drug delivery to be realized. The sonophoresis mode for transdermal drug delivery is able to reduce drug degradation in the liver, stabilize the plasma drug concentration and prevent drug degradation in the digestive tract. Thus, the tissue cell permeability is enhanced and the drug absorption is increased within a short period of time (6).

In the present study, sonophoresis technology was applied in the treatment of TB on the body surface. Anti-TB drugs were administered to the lesions by ultrasonic waves to enable the local lesions to have a higher blood drug concentration compared with the rest of the body, solving the problem of a lower local effective drug concentration. The effective control of TB was consequently assessed by analyzing the improved, cure and efficacy rates. The present study also investigated the role of transdermal ultrasound technology in preventing certain patients from requiring general anesthesia surgery, improving the efficacy of non-surgical treatment and reducing the requirement rate for surgery.

## Materials and methods

### General information

A total of 186 patients were recruited from the Second Department of Thoracic Surgery of Beijing Chest Hospital (Beijing, China) between August 2009 and August 2011. The patients selected for the present study were those with chest wall TB who were treated initially and found to have no rifampicin resistance following a Löwenstein-Jensen medium test. The patients were randomly divided into the transdermal ultrasound, surgery and oral anti-TB drug only groups. There were 62 newly-diagnosed patients in the transdermal ultrasound group (male, 34; female, 28; age range, 13–78 years; mean age, 39 years). The largest and smallest chest wall TB abscesses in this group were 12.0×10.0 cm and 3.0×1.5 cm, respectively. In total, 12 patients were diagnosed with pulmonary TB, while the remaining 50 patients had chest wall TB. The surface surgery treatment group included 62 newly-diagnosed patients with TB (male, 38; female, 24; age range, 17–47 years; mean age, 28.5 years). Of these, 11 patients had pulmonary TB and 51 patients were diagnosed with chest wall TB.

There were 62 newly-diagnosed patients in the oral anti-TB drug only group (male, 41; female, 21 females; age range, 19–71 years; mean age, 43.5 years). In total, eight patients were diagnosed with pulmonary TB, while the remaining 54 patients had chest wall TB. The present study was approved by the Ethics Committee of Capital Medical University, (Beijing, China); written informed consent was obtained from each patient prior to their participation.

### Oral anti-TB drug treatment

The transdermal ultrasound treatment, surgery and oral medication only groups adopted the HREZ anti-TB treatment program, which included isoniazid (H; 300 mg administered orally once per day; Shanxi Yunpeng Pharmaceutical Co., Ltd., Linfen, China), rifampicin (R; 450 mg administered orally once per day; Chongqing Yaoyou Pharmaceutical Co., Ltd., Chongqing, China), ethambutol (E; 750 mg administered orally once per day; Chengdu Jinhua Pharmaceutical Co., Ltd., Chengdu, China) and pyrazinamide (Z; 500 mg administered orally three times per day; Chengdu Jinhua Pharmaceutical Co., Ltd.,). Patients in the oral medication only and transdermal ultrasound groups received anti-TB drugs for 12 months. Patients undergoing surgery received preoperative oral medication for one month prior to surgery and postoperative anti-TB medication for 11 months following surgery. The surgical procedure included tube drainage and debridement of the affected area.

### Transdermal ultrasound treatment

Rifampicin was the transdermal ultrasound-delivered drug. Initially, the patient’s skin was disinfected with povidone-iodine. Based on the Löwenstein-Jensen medium sensitivity test results (only patients without rifacampin resistance were able to take part in ultrasound treatment) a 4×4 cm single piece of cotton dipped in rifampicin solution [5 ml injectable rifampicin aqueous solution (0.3 g)] was spread on the tuberculous abscess. The drug was delivered using an ultrasound transdermal instrument (Beijing Noah Tongzhou Medical Technology Co., Ltd., Beijing, China) for 30 min, with the ultrasound intensity adjusted based on the depth of the lesion (measured by ultrasound). The drug patch remained in place for an additional 1–2 h following each treatment to ensure adequate absorption of the drug.

### Determination of efficacy

The transdermal ultrasound and oral medication only groups were examined by chest B-mode ultrasonography or computed tomography (CT) two months following treatment to confirm the complete disappearance of TB without the appearance of new lesions. Patients were assigned to the: cured category, if there was a full subcision without new lesions; efficacy category, if a size reduction of >60% was observed without new lesions; or improvement category, if a size reduction of >30% and occasional new lesions were observed. The efficacy rate was determined for the cure and efficacy groups. The patients in the surgery group were assessed by chest B-mode ultrasonography and chest CT as part of their routine clinical assessment.

### Case inclusion and exclusion criteria

Patients were included in the study if they met the following criteria: Aged ≥20 years; diagnosed with latent TB by a cervical neck biopsy; treatment-naive with no evidence of a significant cardiac disease; tolerance to ultrasound therapy; and no known allergy to rifampicin.

The exclusion criteria were as follows: Aged <20 years; diagnosed with active pulmonary TB; undergoing cervical lymph node TB retreatment; known allergy to rifampicin; and presence of a significant congenital heart disease that would make ultrasound treatment difficult to tolerate.

### Follow-up period

The follow-up period was 12 months following the transdermal ultrasound treatment, oral anti-TB therapy or surgery.

### Statistical analysis

SPSS 13.0 software (SPSS, Inc., Chicago, IL, USA) was used to analyze the experimental data. Results from the transdermal ultrasound, oral treatment only and surgery groups were compared using the χ^2^ test. P<0.05 was considered to indicate a statistically significant difference.

## Results

### Outcome of the transdermal ultrasound treatment group

**Figure 1:**
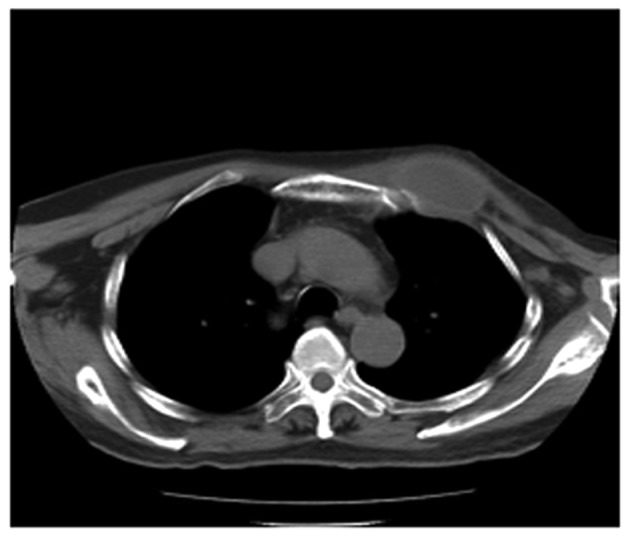
Chest wall tuberculosis prior to transdermal ultrasound treatment.

**Figure 2:**
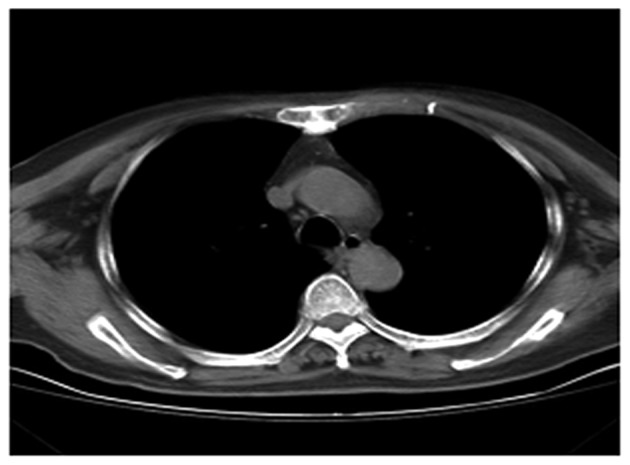
Cured tuberculosis following transdermal ultrasound treatment.

**Figure 3:**
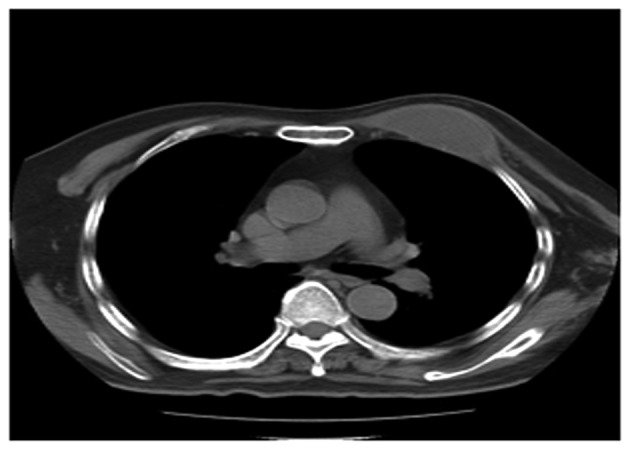
Chest wall tuberculosis prior to transdermal ultrasound treatment.

**Figure 4:**
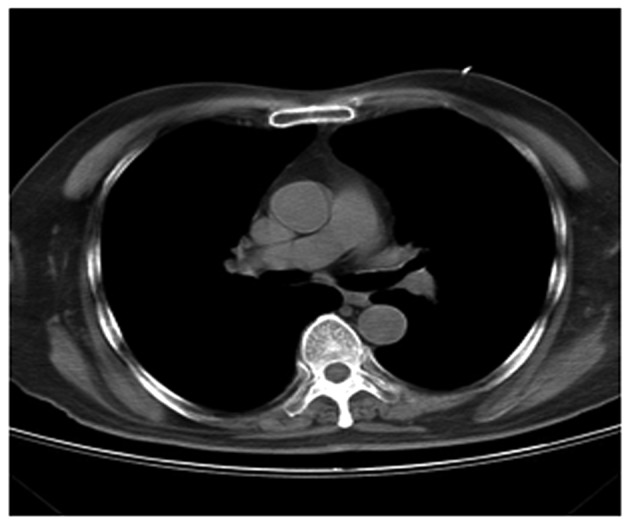
Cured tuberculosis following transdermal ultrasound treatment.

Within the transdermal ultrasound treatment group, 54/62 patients were completely cured, with four patients showing efficacy and two patients showing improvement. The two patients who did benefit from the treatment were hospitalized two weeks later and subsequently discontinued the treatment. The cure rate was 87.10% and the efficiency rate was 93.55% (Figs. 1–4; chest CT scans).

### Outcome of the surgical treatment group

Within the surgery group, 56/62 patients were cured, with four relapses and two patients that suffered from incision wounds that did not heal. As recurrences were observed in four patients, the cure rate was 90.32%. The efficacy rate was not determined in the surgery group it is known to have a high rate of effectiveness (7) and the aim of the present study was to focus on non-invasive treatment options.

### Outcome of the oral drug therapy group

Within the oral drug treatment only group, 12/62 patients were cured. A total of 14 patients were classified into the efficacy category, while eight patients demonstrated marked improvement; 28 patients did not show any improvement. The cure rate was 19.35% and the efficiency rate was 41.93%.

### Statistical significance

No statistically significant difference was observed in the cure rate between the transdermal ultrasound and surgery groups (Table I). However, a statistically significant difference was identified in the cure rate between the transdermal ultrasound and oral drug only groups (Table II). The cure and efficiency rates were significantly higher in the transdermal ultrasound group when compared with the oral treatment only group (Table III).

## Discussion

TB is a chronic infectious disease caused by *M. tuberculosis*. According to World Health Organization statistics, ~1/3 of the world population is infected with *M. tuberculosis* (1). Of the TB cases and TB mortalities, 95 and 98%, respectively, occur in developing countries. The TB infection rate in China is 44.5%, with an estimated 550 million individuals infected with *M. tuberculosis* (1,8).

The majority extrapulmonary TB cases are the result of a lung infection by *M. tuberculosis*. The incidence rate of extrapulmonary TB is an important contributor to the TB epidemic. Although extrapulmonary TB accounts for only 10% of the total number of TB cases, the infection can exist in numerous parts of the body with different infection routes and symptoms. As a result, the diagnoses, examination methods and treatments also differ (9).

Body surface TB includes chest wall TB and lymphatic TB, the two of which are common types of extrapulmonary TB. Chest wall TB is the most common type of chest wall disease, particularly among individuals aged <30 years (10,11). Typical symptoms of chest wall TB include abscesses and chronic sinus formation (12), which are often secondary to lung, pleura or mediastinal TB. The majority of patients do not experience symptoms or have mild pain, although abscesses may rupture spontaneously or eventually form chronic open sinuses.

Since chest wall TB exhibits no clinical symptoms in the majority of patients with the early form of the disease, chest wall TB frequently has multiple variants and affects a large percentage of the population (13). When chest wall TB is diagnosed, abscesses and sinuses have already formed or ruptured. Since blood vessels adjacent to the chest wall TB area are damaged and *M. tuberculosis* resides within the lymph nodes (lymph nodes have intact membranes), it is difficult for anti-TB drugs to achieve an effective concentration in the system. Therefore, systemic treatment with anti-TB drugs often does not achieve desirable results and long-term anti-TB drug treatment is frequently combined with surgery (14,15). Surgical treatment is able to completely remove the necrotic lesions and *M. tuberculosis*. However, since chest wall TB can affect multiple locations, the surgical approach may be difficult. Surgical procedures for chest wall TB vary based on the condition of the patient, and often require a rib resection and a muscle flap (16). Such surgeries generally require intravenous anesthesia and endotracheal intubation, and the affected area is significant. Thus, elderly patients and patients in a poor physical condition are often unable to tolerate surgery. During surgery, the lesions must be thoroughly cleaned, along with the removal of all the tuberculous tissue. In addition to the surgical removal of the skin and subcutaneous soft tissue, a rib resection, partial thoracoplasty, clavicle resection and a pleurectomy are performed. The wound is subsequently bandaged and compressed for a long period of time, and poor healing may often lead to early infection. Due to the large postoperative skin damage and slow-healing of the wounds, tension reduction or free flap incisions are often used, which can cause serious and unsightly scars (3,17).

The recurrence rate from chest wall TB surgery is high. Although there have been significant improvements in preoperative diagnosis, debridement and surgical techniques, due to the low concentration of anti-TB drugs in the tuberculous area and the poor tolerance of oral anti-TB drugs by certain patients, the recurrence rate of TB remains at 7–8% (12). The high recurrence rate also reflects other factors, including the residual TB tissue, patients with diabetes and drug-resistant TB strains. In certain cases, complete curative effects are difficult to achieve even following multiple surgeries; thus, chest wall TB is a debilitating disease.

Transdermal ultrasound-mediated drug delivery is a targeted drug delivery technology. The technique takes advantage of the principle of high-frequency electromagnetic fields and the closed-circuit nature of biological tissues to deliver a drug quickly and directly toward the diseased areas, increasing tissue cell permeability (18). Since skin functions as a natural barrier, the majority of drugs, even low-dose, highly efficacious drugs, are unable to permeate the skin sufficiently to meet the treatment requirements. Thus, overcoming the skin barrier and promoting drug penetration to achieve a therapeutic transdermal concentration is one of the key challenges in transdermal drug delivery research (19,20). Electromagnetic field effects have been demonstrated to activate drug activity and efficacy (21). The drug is introduced into the body without damage to the skin, pain, gastrointestinal irritation or other side-effects. Currently, significant progress has been made to use ultrasound-mediated physical therapy to increase the sensitivity of localized chemotherapy and radiation in the treatment of cancer, alleviate pain from cancer and improve the synergy between frozen and heat therapy (22,23). However, to the best of our knowledge, the present study introduced transdermal ultrasound-mediated anti-TB drug delivery for the treatment of chest wall TB for the first time. The outcome was superior to that of oral anti-TB only treatment, and the procedure did not require surgical intervention for a number of patients.

In conclusion, the current study demonstrated that transdermal ultrasound technology may be applied for the treatment of body surface TB. Ultrasonography quickly and directly delivered the anti-TB drugs to the diseased area and achieved a high local plasma concentration. The transdermal ultrasound method overcame the low local drug concentration problem and was demonstrated to be an effective method to control TB. As a result, a number of patients may no longer have to undergo surgery with general anesthesia. The efficacy of this new method was significantly improved compared with the administration of oral anti-TB drugs only. Therefore, transdermal ultrasound-mediated drug delivery provides an important non-surgical therapeutic option for patients with chest wall TB.

## Figures and Tables

**Table I:** Cure rate comparison between the transdermal ultrasound and surgery groups.

Group (n)	Total cases (n)	Cured cases (%)	Cure rate
Transdermal ultrasound	62	54	87.10
Surgery	62	56	90.32
P-value	-	-	0.57

**Table II:** Cure rate comparison between the transdermal ultrasound and oral medication only groups.

Group	Total cases (n)	Cured cases (n)	Cure rate (%)
Transdermal ultrasound	62	54	87.10
Oral medication	62	12	19.35
P-value	-	-	<0.01

**Table III:** Efficacy rate comparison between the transdermal ultrasound and oral medication only groups.

Group	Total cases (n)	Efficacy cases (n)	Efficacy rate (%)
Transdermal ultrasound	62	58	93.55
Oral medication	62	26	41.93
P-value			<0.01
